# Influence of Weight Gain Rate on Early Life Nutritional Status and Body Composition of Children

**DOI:** 10.1155/2014/616108

**Published:** 2014-11-04

**Authors:** Sarah Aparecida Vieira, Taís Cristina Araújo Magalhães, Andréia Queiroz Ribeiro, Silvia Eloiza Priore, Sylvia do Carmo Castro Franceschini, Luciana Ferreira da Rocha Sant'Ana

**Affiliations:** Department of Nutrition and Health, Federal University of Viçosa, 36570-000 Viçosa, MG, Brazil

## Abstract

*Objective*. To evaluate the influence of the weight gain rate at 4–6 months on nutritional status and body composition in children between 4 and 7 years of age. *Methods*. Retrospective cohort study, sample of 257 children. Data collection was performed in two stages, with the first relating to retrospective data of weight gain from birth to the first 4–6 months of life in the patient records. Measurements of weight, height, waist circumference, and body composition in children between ages 4 and 7 years were obtained. Nutritional status was assessed by the BMI/age. Control variables, such as pregnancy, breastfeeding, lifestyle, and sociodemographics, were studied. Descriptive analysis and multiple linear regression were performed. *Results*. In the nutritional status assessment, the prevalence of overweight observed was 24.9%. After adjusting for control variables, it was found that the increase of the WGR at 4–6 months of age explained the occurrence of higher BMI/age, percentage of total body fat, body fat percentage in the android region, and waist circumference in children between 4 and 7 years of age. *Conclusion*. The increase of the WGR in the first months of life can lead to the occurrence of higher values of parameters of nutritional status and body composition in later life.

## 1. Introduction

In recent decades a rapid increase in the prevalence of overweight has been observed in both the developed and the developing countries, and it is considered a chronic and epidemic disease related to high morbidity and mortality rates [1, 2].

According to the Household Budget Survey conducted in 2008-2009, in Brazil overweight affects 33.5% of children aged five to nine years, with 16.6% of boys being obese, and among girls, obesity has reached 11.8% [3].

Excess weight and body fat are associated with the development of various morbidities, such as cardiovascular diseases, hypertension, diabetes, hypercholesterolemia, and some cancers [4]. Moreover, obese people, particularly children and adolescents, often have low self-esteem, affecting school performance and social interaction [5].

Risk factors for the development of obesity and excess body fat have been identified at different stages of life, especially when there is a greater acceleration of growth early in life [6]. Studies show a relationship between the growth rate in the first months of life and the risk of obesity, excess body fat, insulin resistance and lifelong cardiovascular diseases [7–9]. Others have shown that excessive weight gain in early life can negatively affect health in the future, and evidence suggests that children with low birth weight with a rapid weight recovery in the first months of life have subsequently an increased prevalence of excess weight [9, 10]. After following French children from birth to adolescence, Botton et al. [11] observed a relationship between higher velocity of weight gain in the first months of life and changes in nutritional status and body composition in adolescence.

The association between higher speed of weight gain in early life and development of cardiovascular risk factors in later ages indicates that existing systems for monitoring child growth can adopt this criterion for identifying children at higher risk for cardiovascular disease throughout life [12].

In Brazil, there are few studies evaluating the relationship between growth in early life and development of changes in nutritional status and body composition in later ages [13, 14]. Thus, this study evaluated the influence of the weight gain rate at 4–6 months on nutritional status and body composition of children aged between 4 and 7 years in Viçosa, Minas Gerais, Brazil.

## 2. Methods

### 2.1. Study Design and Population

This is a retrospective cohort study, whose sample consisted of children from 4 to 7 years of age accompanied by a Lactation Support Program (PROLAC) in the first months of life in Viçosa, Minas Gerais, Brazil.

PROLAC is an Outreach Program of the Federal University of Viçosa (UFV) in partnership with the Human Milk Bank of the city that began in 2003. Among its activities it conducts orientations to the mother during the postpartum period with a view to promote breastfeeding and nutritional care for breastfeeding mothers and children in their first year of life.

The initial study sample was made of 371 children, which includes all those who initiated the monitoring at PROLAC between August 2003 (date of the beginning of the program) and November 2007 (determined period so that they would have in the beginning of the study the minimum age of 4 years) and that fulfilled the inclusion criteria.

The inclusion criteria considered in the first stage of the study were having a record of the weight and length at birth, weight and length at least at the fourth month of life, having been born at term, not being born with low birth weight or macrosomia, and presence of identification data that would allow their location [15, 16]. As exclusion criteria, we considered the presence of health changes reported in the medical records that could influence nutritional status in early life.

Three attempts to locate children were made through home visits and searches for new addresses in the case of moves. However, 78 children were excluded from the study for failure to locate them.

The inclusion criteria considered in the study after locating the children were the written consent of the parent or guardian and the conducting of all phases of the study. As exclusion criteria, we considered the presence of disease or use of medications that might interfere with nutritional status or body composition at 4–7 years of age. These criteria generated losses in the study and were represented by children who had health changes and/or use of medications that interfered with the assessment of nutritional status (7 children), mother or guardian's refusal to participate in the study (12 children), and not performing all stages of the study (17 children). Thus, the final sample consisted of 257 children, representing 69.3% of the initial sample.

### 2.2. Study Variables

Data collection was performed in two stages: the collection of retrospective data in the PROLAC records and children's data relating to the ages evaluated in the study (4–7 years).

### 2.3. Main Exposure Variable

To calculate the weight gain rate of children, data of weight at birth and at 6 months were compared. In cases of those who were not assessed at that age, the measurements obtained at ages 4 or 5 months were considered:

(1) weight  gain  rate  (grams/day)  =(weight  at  4–6  months−birth  weight)   ÷age  in  days.

### 2.4. Outcome Variables

Children between the ages of 4 and 7 years were evaluated for weight and height, adopting techniques proposed by Jelliffe [17]. The percentage of total body fat and of fat in the android region (representing the abdominal fat) was assessed by DEXA (dual energy X-ray absorptiometry) and the waist circumference was measured at the level of the umbilical scar.

To assess nutritional status of the Body Mass Index by age (BMI/A), anthropometric references from the World Health Organization (WHO) were adopted [18, 19]. To obtain the index in* z*-score, the software WHO Anthro Plus was used, and the diagnosis of the nutritional status of the children was carried out as recommended by WHO [20, 21].

### 2.5. Control Variables

To verify the independent influence of the VGP in the first months of life in the nutritional status and body composition at 4–7 years of age, the following control variables were considered: pregnancy, birth and breastfeeding (obtained from the PROLAC records), and sociodemographic characteristics, lifestyle, and current eating habits through the use of questionnaires to the children's mothers or guardians. The lifestyle habits were obtained by using an adapted Andaki questionnaire [22].

The information on the number of prenatal appointments, prepregnancy nutritional status, and weight gain during pregnancy was obtained from the prenatal card of the pregnant mother. Weight gain during pregnancy was categorized according to the classification of the Institute of Medicine [23], considering the prepregnancy BMI.

The diet variables were obtained by applying 3 Food Records on nonconsecutive days, including a weekend day, supplemented with information from food consumed at school. Analyses of the food records were performed using the Diet Pro version 5.1 software.

The estimated energy requirement (EER) was calculated using factors of physical activity estimated according to the life style questionnaire [24]. We compared the value of EER and mean energy intake of the three days, obtained by dietary records, to determine the energy balance variable. The difference between the energy intake average and the EER value greater than two standard deviations of the need was considered a positive energy balance [25].

## 3. Statistical Analyses

The analyses were conducted in the Social Package Statistical Science (SPSS) for Windows version 11.0 and STATA version 9.1.

The sample characterization was performed using frequency distribution and estimated measures of central tendency and dispersion. The Shapiro-Wilk test was used to verify the distribution of the variables in relation to the expected in the Gauss curve. To verify the correlation between continuous variables the Spearman or the Pearson correlation tests were used, and to compare groups the Mann-Whitney or the Student's *t*-test was used.

Multiple linear regression analysis was performed to evaluate the effect of independent variables on the dependent ones (nutritional status and body composition). The criteria set for inclusion of variables in multiple linear regression was the relationship with the dependent variable in the analysis of simple linear regression, considering a value of *P* < 0.20.

The suitability and fit of the linear regression model, the normality of residual distribution, and the presence of heteroscedasticity were evaluated. As an indicator of multicollinearity, the variance inflation factor was used in the multiple regression analyses.

Outcome variables that were not normally distributed were subjected to logarithmic transformation for inclusion in linear regression analyses. The level of significance used was *α* < 5%.

### 3.1. Ethical Aspects

The project was approved by the Ethics Committee on Human Research of the Federal University of Viçosa (Reference number 094/2011).

All children participating in the study had received individualized nutritional care, with delivery of the evaluation and nutritional counseling results being referred, when necessary, to consultation with a pediatrician.

## 4. Results

The sample consisted of 257 children, with 142 (55.2%) being males. The average age was 71.5 (SD = 12.5) months.

When comparing children evaluated at 4–7 years old with those who were not included in the study (*n* = 144), no statistically significant differences were observed in relation to the median WGR (*P* = 0.91), practice of AME or predominant (*P* = 0.13), and mean age at the beginning of the study (*P* = 0.86). Only significant difference was observed in relation to gender (*P* = 0.01), since the percentage of female children (60.5%) was higher than that of male children (39.5%) in the sample of those who represented the study losses. In the sample of children assessed at 4–7 years of age, the prevalence of male children was higher.

The WGR median in the first months of life observed for males was 26.3 g/day and 22.8 g/day for females (*P* < 0.001). The assessment of the nutritional status of children 4–7 years old by BMI/A showed the following results: 2 children (2.7%) were underweight, 186 (72.4%) were normal weight, 8 (3.1%) were at risk of overweight, 34 (13.2%) were overweight, 18 (7.0%) were obese, and 4 (1.6%) were with severe obesity. When considering the categories risk of overweight, overweight, obesity, and severe obesity, the prevalence of overweight observed was 24.9%.

The WGR at 4–6 months had a positive correlation with all assessed outcomes (*P* < 0.001); that is, the higher the WGR at 4–6 months the higher the BMI/A, the percentage of body fat, the percentage of body fat in the android area, and the waist circumference at ages between 4 and 7 years (Table 1).

Children whose mothers had higher prepregnancy BMI had higher BMI/A, waist circumference, and body fat percentage in the android region of children between 4 and 7 years of age. In addition, children with higher birth weight had higher BMI/A and waist circumference, and children with higher birth length showed higher values of waist circumference in the evaluated ages. The current age in months also correlated to the percentage of total body fat, the android region, and waist circumference, whereas older children showed higher values for these outcomes.

In the comparison of means and medians of the evaluated outcomes with the variables of gestational control and birth, referring to the practice of breastfeeding and sociodemographics, children whose mothers had an excessive weight gain during pregnancy had higher median BMI* z*-score/A, percentage of total body fat and of body fat in the android region, and waist circumference between 4 and 7 years of age (Table 2). Furthermore, between the genders, differences in the means of BMI/A were observed, where male children had higher average for this outcome. The female children had higher median percentage of total body fat and of body fat in the android region in the evaluated ages.

In comparisons of means and medians of outcomes with the control variables related to lifestyle and nutrition of children (Table 3), children who performed active activities for one hour or more per day had a lower median percentage of body fat of children between 4 and 7 years of age when compared to those who devoted less than one hour per day on these activities. Another variable with a significant result was the daily time in light activities (usually sitting). Children who spent an hour or more per day on these activities had higher median percentage of body fat when compared to those who carried out these activities in a time equal to or less than one hour daily.

Tables 4, 5, and 6 are the results of the simple linear regression analyses for the assessed outcomes, according to the control variables of the study.

The variables with *P* < 0.20 in the simple linear regression analyses were included in the multiple regression models. The results showed that the increasing of the WGR between 4 and 6 months of age explained the occurrence of higher values of BMI* z*-score/A, percentage of total body fat and of body fat in the android region, and waist circumference of children between 4 and 7 years of age, after adjustment for the control variables (Table 7).

It is noteworthy that other variables in the multivariate analysis, besides the WGR, were independently associated with BMI/A (prepregnancy BMI, weight at birth, and place of residence), body fat percentage (prepregnancy BMI, weight gain during pregnancy, daily active time, sex, and current age), activities, percentage of fat in the android region (weight gain during pregnancy, gender, and current age), and waist circumference (prepregnancy BMI, weight gain during pregnancy, weight at birth, and current age).

Assessments of the linear regression analysis adjustment showed adequacy of models.

## 5. Discussion

The WGR at 4–6 months of age explained the variation of all outcomes of nutritional status and body composition of children between 4 and 7 years of age, with its increase related to higher BMI/A, percentage of total body fat and of fat in the android region, and waist circumference. This relationship was maintained in the multiple linear regression analyses, after adjusting by the control variables.

In other investigations a greater weight gain in childhood was also associated with changes in nutritional status in later ages [10, 26]. In an American longitudinal study children of different ethnicities evaluated during the first six months and at four years of age, an increased weight gain rate in the first months of life increased the risk of overweight at a later age (OR = 1.43; 95% CI: 1.27–1.60) [26].

The Goodell et al. [27] study with American children in the first three years of life, from low-income families, showed that those with a greater weight gain in the first year had 9.24 times higher risk (OR = 9.24, 95% CI: 3.73–22.91) to become obese even at the beginning of preschool. These results confirm the findings of a systematic literature review conducted by Ong and Loos [12], who observed a positive association between accelerated weight gain in the first two years of life and risk of obesity later in life in the 21 studies analyzed.

In our study the percentage of fat in the android region and the waist circumference at 4–7 years of age were measured for evaluation of localized fat in the abdominal region [16, 28]. This fact is of great importance in view of the importance of evaluating the accumulation of abdominal fat in children, because studies have shown that excess fat in this region is associated with the development of various morbidities such as hypertension, diabetes, and cardiovascular disease throughout life [29, 30].

In a study conducted in France a higher weight gain rate at 3 months of age was associated with increased values of waist circumference and body fat in adolescence (*P* < 0.001) [11].

Nutritional assessment of children for BMI by age showed a prevalence of underweight at 4–7 years of age of 2.7% and of overweight of 24.9% of the assessed children, showing the process of nutritional transition, which occurs throughout the world, characterized by the reduction of the deficit and the increase of overweight [1, 31]. In the study by Gigante et al. [32] in Pelotas, RS, by comparing birth cohorts of 1982 and 1993, there was an increase in the prevalence of overweight about two times higher in children born in 1993 when compared to those in a similar age born in 1982. On the other hand, there was a decrease in the prevalence of stature deficit of nearly 50%, comparing the same children in both periods. This trend was also seen in the results of the latest Household Budget Survey (HBS) [3].

Although not goals of this paper, interesting associations were observed between the various control variables assessed and the outcomes of nutritional status and body composition between age 4 and 7 years. Variables such as prepregnancy BMI, weight gain during pregnancy, birth weight, daily time on active activities, and frequency of consumption of some foods high in sugars and fats were independently associated with the outcomes. These results highlight the multiple causality of alterations of nutritional status and body composition in childhood [33, 34].

No children with low birth weight (<2500 g) and preterm (<37 weeks gestation) were evaluated in order to investigate the relationship between the weight gain rate and length during the first months of life and outcomes assessed at later ages independent of the catch-up growth that is observed in children born preterm and/or with low birth weight [35]. Birth weight was included in the analyses as a control variable of the study and it was observed that in multivariate analyses, after adjustment for other factors, birth weight was associated with increased BMI for age and waist circumference for 4–7 years of age. This result was similar to that seen in other studies, which suggested that children born with lower weight would be more protected against changes in nutritional status in childhood [36, 37].

Among children participating in the study, only 19% were exclusively or predominantly breastfed from 4–6 months of age, with this prevalence being similar to that observed in children aged 4 to 5 months participating in a cohort study conducted in five regions of Brazil [38]. Since these children were followed in the first months of life in a program that had supporting and encouraging breastfeeding as its main objective, it was expected to find a higher prevalence of this practice. In the present study, no association was observed between breastfeeding and the outcomes of nutritional status and body composition at 4–7 years of age, but other studies have shown a protective effect of breastfeeding against nutritional and health alterations throughout life [39, 40].

It has been demonstrated that breastfeeding may influence the weight gain rates and length during the first months of life, differences in growth patterns of breastfed children and those fed by formulas being observed [41, 42]. Thus, to obtain independent association between weight gain rates and length during the first months and the outcomes assessed at 4–7 years of age, the practice of breastfeeding was a variable considered in the analyses as a control variable of the study.

It is worth noting as a positive point of this study the large number of control variables investigated (gestational, birth, sociodemographics, lifestyle, and eating habits), which could relate to nutritional status and body composition at childhood. Thus, it was possible to assess the independent effect of the weight gain rate on the outcomes evaluated.

Although the influence of energy intake on nutritional status and body composition of individuals is already established, no relationship between energy balance and evaluated parameters was observed [43]. What can explain this observation is the existence of errors inherent in the method used to assess dietary intake, such as the difficulty to write down the foods and estimating portions consumed [44].

Another positive aspect of the study to be considered was the way of obtaining retrospective data. Gestational information, birth and weight gain at 4–6 months, and breastfeeding were obtained from well-structured records from established care protocols, ensuring the reliability of the data obtained. Many studies collect data on recalled weight and length at birth and breastfeeding [14, 45], given that discrepant results can be observed among studies using recorded data and those assessing recalled data, the latter being subject to recall bias [46].

As a limitation of the present study, there is the fact of no possibility of achieving a sample calculation that was representative of the population of Viçosa, because the recorded data were obtained from a program that meets a portion of the city population. To minimize this effect, all children served by the program who met the inclusion criteria were included in the study. In addition, another limitation, inherent in every longitudinal study, was the losses throughout the study. Since these identification data were obtained in the past, many children were not located. Nevertheless, the losses did not affect the representativeness of the sample, since, except for gender, other variables did not differ from the group analyzed.

## 6. Conclusion

In the present study the influence of the weight gain rate in the first 4–6 months of age on the nutritional status and body composition between ages 4 and 7 years was observed, and the increase of this rate explained higher values of BMI by age and percentage of total body fat and of fat in the android region, and waist circumference among the assessed children.

The association between increased weight gain rate in the first months of life and the occurrence of higher values of nutritional status and body composition parameters in later ages suggests that this criterion can be used for monitoring child growth systems by identifying children with higher risk of changes in nutritional status and body composition throughout life. Thus, early interventions may be undertaken with a goal to provide adequate control of nutritional status and body composition in childhood.

## Figures and Tables

**Table 1:** Correlation between Body Mass Index for age, body fat percentage, fat percentage of the android region, and waist circumference with rates of weight and length gain and control variables of children 4–7 years of age, Viçosa, MG, 2011-2012.

Variables	BMI/A (*z*-score)		Body fat %		Fat % in the android region		Waist circumference (cm)	
	*r*	Value of *P* ^a^	*r*	Value of *P* ^b^	*r*	Value of *P* ^b^	*r*	Value of *P* ^b^
Weight gain rate (g/day)	0.37	**<0.001**	0.22	**<0.001**	0.20	**<0.001**	0.37	**<0.001**
Pregestational BMI^1^ (kg/m^2^)	0.27	**<0.001**	0.12	0.05	0.16	**0.01**	0.21	**<0.001**
Birth weight (kg)	0.17	**0.01**	0.02	0.70	0.01	0.81	0.20	**<0.001**
Length at birth (cm)	0.10	0.08	0.02	0.70	0.02	0.67	0.12	**0.04**
Current age (months)	0.10	0.11	0.15	**0.02**	0.14	**0.02**	0.40	**<0.001**
Maternal age^2^ (years)	−0.01	0.88	0.02	0.68	−0.02	0.77	0.05	0.40
*Per capita* income (in reais)	0.06	0.33	0.09	0.16	0.01	0.87	0.04	0.53

BMI = Body Mass Index; % = percentage; cm = centimeter; g = gram; kg = kilogram; m = meter; *r* = correlation coefficient; ^a^Pearson correlation; ^b^Spearman correlation; ^1^ *n* = 249; ^2^ *n* = 256.

**Table 2:** Comparison of Body Mass Index for age, body fat percentage, fat percentage of the android region, and waist circumference according to gestational variables, birth, and breastfeeding and sociodemographics of children 4–7 years of age, Viçosa, MG, 2011-2012.

Variables	IMC/I	Body fat	Fat in the android region	Waist circumference
	Mean (SD)	Median (min–max)	Median (min–max)	Median (min–max)
	(*z*-score)	(%)	(%)	(cm)
Number of prenatal visits				
≥6 (*n* = 144)	0.16 (1.21)	15.90 (5.20–42.50)	8.45 (4.00–44.40)	53.97 (42.80–83.00)
<6 (*n* = 105)	0.24 (1.34)	15.50 (6.50–38.90)	10.40 (4.00–39.10)	55.70 (46.50–83.20)
*P* value	0.63	0.58	0.10	0.12
Pregnancy weight gain				
Not excessive (*n* = 178)	0.04 (1.10)	15.45 (6.30–39.50)	8.35 (4.00–33.80)	53.95 (45.00–83.20)
Excessive (*n* = 59)	0.61 (1.57)	19.80 (5.20–42.50)	12.10 (4.00–44.40)	55.80 (47.10–83.00)
*P* value	**0.01**	**0.01**	**0.01**	**0.03**
EPB				
Yes (*n* = 49)	0.21 (1.35)	17.60 (6.50–42.50)	9.60 (4.00–44.40)	52.55 (47.20–83.20)
No (*n* = 208)	0.18 (1.25)	15.70 (5.20–40.50)	8.75 (4.00–39.10)	54.60 (42.80–77.60)
*P* value	0.88	0.57	0.69	0.18
Gender				
Male (*n* = 142)	0.36 (1.35)	13.55 (5.20–40.50)	7.50 (4.00–41.90)	54.70 (42.80–83.20)
Female (*n* = 115)	−0.01 (1.13)	18.60 (8.60–42.50)	11.20 (4.00–44.40)	53.85 (45.00–83.00)
*P* value	**0.02**	**0.00**	**0.00**	0.07
Maternal education (years)				
>8 years (*n* = 150)	0.25 (1.29)	16.10 (6.30–40.50)	8.85 (4.00–41.90)	54.65 (42.80–83.20)
≤8 years (*n* = 120)	0.13 (1.23)	15.30 (5.20–42.50)	8.90 (4.00–44.40)	54.00 (45.0–83.0)
*P* value	0.47	0.43	0.72	0.52
Paternal education (years)				
>8 years (*n* = 105)	0.14 (1.30)	15.70 (6.50–42.50)	7.80 (4.00–44.40)	53.85 (42.80–83.00)
≤8 years (*n* = 129)	0.27 (1.22)	16.60 (6.30–36.00)	9.40 (4.00–39.10)	55.40 (45.00–83.20)
*P* value	0.42	0.82	0.57	0.05
*P* value	0.83	0.41	0.32	0.55
Place of residence				
Rural (*n* = 23)	0.54 (1.22)	18.70 (6.40–35.10)	7.70 (4.00–32.60)	55.70 (48.30–83.20)
Urban (*n* = 234)	0.15 (1.27)	15.75 (5.20–42.50)	9.10 (4.00–44.40)	54.00 (42.80–83.00)
*P* value	0.16	0.39	0.77	0.38

BMI = Body Mass Index; min = minimum; max = maximum; cm = centimeter; SD = standard deviation; EPB = exclusive or predominant breastfeeding. *P* values from the Mann-Whitney test and Student *t* test (for BMI/A).

**Table 3:** Comparison of Body Mass Index by age, body fat percentage, percentage of body fat in the android region, and waist circumference with variables in daily habits and nutrition of children between 4 and 7 years of age, Viçosa, MG, 2011-2012.

Variables	BMI	Body fat	Fat in the android region	Waist circumference
	Average (DP)	Median (min–max)	Median (min–max)	Median (min–max)
	(escore-*z*)	(%)	(%)	(cm)
Hours of TV				
≤2 (*n* = 113)	0.07 (1.23)	15.70 (6.40–36.00)	8.60 (4.00–32.60)	54.00 (45.0–83.20)
>2 (*n* = 144)	0.28 (1.29)	16.05 (5.20–42.50)	9.15 (4.00–44.40)	54.55 (42.80–83.00)
Value *P*	0.17	0.12	0.33	0.11
Physical activity time^1^ (hours)				
>1 (*n* = 96)	0.04 (1.20)	14.60 (5.20–35.10)	8.30 (4.00–35.20)	53.55 (42.80–83.20)
≤1 (*n* = 161)	0.28 (1.30)	16.60 (6.50–42.50)	9.10 (4.00–44.40)	55.00 (45.00–83.00)
Value *P*	0.15	**0.01**	0.42	0.07
Light physical activity time^2^ (hours)				
≤1 (*n* = 158)	0.20 (1.27)	14.60 (5.20–42.50)	8.65 (4.00–44.40)	54.05 (42.80–83.20)
>1 (*n* = 99)	0.16 (1.27)	16.90 (6.40–38.90)	9.40 (4.00–38.50)	54.30 (46.45–77.60)
Value *P*	0.81	**0.02**	0.33	0.56
Sports Activity				
Yes (*n* = 21)	0.58 (1.27)	18.80 (6.50–37.90)	11.20 (4.00–41.90)	56.10 (47.20–74.60)
No (*n* = 236)	0.15 (1.26)	15.70 (5.20–42.50)	8.80 (4.00–44.40)	54.00 (42.80–83.20)
Value *P*	0.13	0.21	0.85	0.09
Energy balance				
Not positive (*n* = 194)	0.22 (1.27)	15.90 (6.30–42.50)	9.10 (4.00–44.40)	54.47 (42.80–83.00)
Positive (*n* = 63)	0.10 (1.25)	15.80 (5.20–36.00)	8.80 (4.00–39.10)	53.55 (45.00–83.20)
Value *P*	0.50	0.33	0.66	0.40

BMI = Body Mass Index; TV = television; min = minimum; max = maxim; cm = centmeter; SD = standard deviatio; ^1^play ball, ride bikes, run, and so forth; ^2^play with dolls, cars, house, homework, and so forth. Value of *P* derived from the Mann-Whitney test and Student *t* test (for BMI/I).

**Table 4:** Simple linear regression coefficients and respective confidence intervals for the association between Body Mass Index by age, body fat percentage, percentage of fat in the android region, and waist circumference between 4 and 7 years of age, according to variables in the speed of weight gain, maternity, gestational, birth, and breast feeding of children in Viçosa, MG, 2011-2012.

Variables	BMI/I (escore-*z*)			Body fat (%)^*^			Fat in android region (%)^*^			Waist circumference^*^ (cm)		
	*β*	IC 95%	*P* value	*β*	IC 95%	*P* value	*β*	IC 95%	*P* value	*β*	IC 95%	*P* value
VGP (g/dia)	0.09	0.06–0.12	**<0.001**	0.01	0.01–0.03	**<0.001**	0.02	0.01–0.04	**<0.001**	0.01	0.00-0.01	**<0.001**
Number of prenatal visits												
≥6	—	—	—	—	—	—	—	—	—	—	—	—
<6	0.07	−0.24–0.40	0.63	0.03	−0.07–0.14	0.52	0.13	−0.02–0.29	0.10	0.03	−0.00–0.05	0.06
Pregestational BMI (kg/m^2^)	0.07	0.03–0.11	**<0.001**	0.01	−0.00–0.02	0.09	0.02	0.00–0.04	**0.03**	0.01	0.00-0.01	**0.01**
Gestational weight gain (kg)												
Not Excessive	—	—	—	—	—	—	—	—	—	—	—	—
Excessive	0.57	0.20–0.93	**<0.001**	0.16	0.04–0.28	**0.01**	0.29	0.10–0.47	**<0.001**	0.04	0.01–0.07	**0.01**
Birth weight (kg)	0.01	0.00-0.01	**<0.001**	0.00	−0.00-0.00	0.62	0.00	−0.00-0.00	0.58	0.00	0.00-0.00	**0.01**
Birth length (cm)	0.08	−0.01–0.17	0.08	0.01	−0.02–0.04	0.47	0.02	−0.03–0.06	0.46	0.01	−0.00–0.01	0.06
AMEP												
Yes	—	—	—	—	—	—	—	—	—	—	—	—
No	−0.03	−0.42–0.36	0.88	−0.03	−0.16–0.0	0.60	−0.04	−0.24–0.15	0.66	0.00	−0.03–0.04	0.82

VGP = speed of weight gain logarithm; VGC = speed of length; BMI = Body Mass Index; *β* **= **linear regression coefficient; IC = confidence interval; cm = centimeter; g = gram; ^*^in logarithm.

**Table 5:** Simple linear regression coefficient and respective confidence intervals for the association between Body Mass Index by age, body fat percentage, fat percentage in the android region, and waist circumference between 4 and 7 years of age, according to sociodemographics, Viçosa, MG, 2011-2012.

Variables	BMI/I (escore-*z*)			Body fat (%)^*^			Fat in android region (%)^*^			Waist circumference^*^ (cm)		
	*β*	IC 95%	*P* value	*β*	IC 95%	*P* value	*β*	IC 95%	*P* value	*β*	IC 95%	*P* value
Sex												
Masculine	—	—	—	—	—	—	—	—	—	—	—	—
Feminine	−0.37	−0.68–−0.62	**0.02**	0.24	0.14–0.34	**0.00**	0.24	0.08–0.39	**<0.001**	−0.02	−0.05–0.01	0.14
Actual age (months)	0.01	−0.00–0.02	0.14	0.01	0.00-0.01	**0.02**	0.01	0.00-0.01	**<0.001**	0.00	0.00-0.01	**<0.001**
Maternal age (years)	−0.01	−0.03–0.02	0.55	0.00	−0.01–0.01	0.84	0.00	−0.01–0.01	0.89	0.00	−0.00-0.00	0.38
Maternal Education												
>8 years	—	—	—	—	—	—	—	—	—	—	—	—
≤8 years	−0.11	−0.43–2.20	0.47	−0.04	−0.15–0.06	0.39	−0.02	−0.18–0.13	0.76	−0.01	−0.03–0.02	0.67
Paternal Education												
>8 years	—	—	—	—	—	—	—	—	—	—	—	—
≤8 years	0.13	−0.19–0.45	0.42	0.00	−0.10–0.11	0.93	0.04	−0.12–0.20	0.63	0.02	−0.00–0.05	0.09
*Per capita* Income (reais)	0.00	−0.00-0.00	0.45	0.00	−0.00-0.00	0.37	−0.00	−0.00-0.00	0.76	0.00	−0.00-0.00	0.70
Place of residence												
Rural	—	—	—	—	—	—	—	—	—	—	—	—
Urban	−0.38	−0.93–0.15	0.16	−0.06	−0.24–0.12	0.50	0.05	−0.22–0.33	0.70	−0.02	−0.07–0.02	0.30

BMI = Body Mass Index; *β* **= **linear regression coefficient; IC = confidence interval, cm = centimeter; ^*^in logarithm.

**Table 6:** Simple linear regression coefficient and respective confidence intervals for the association between Body Mass Index, body fat percentage, percentage of fat in the android region, and waist circumference between 4 and 7 years of age, according to variables in lifestyle and eating habits, Viçosa, MG, 2011-2012.

Variables	BMI/I (escore-*z*)			Body fat (%)^*^			Fat in android region (%)^*^			Waist circumference^*^ (cm)		
	*β*	IC 95%	*P* value	*β*	IC 95%	*P* value	*β*	IC 95%	*P* value	*β*	IC 95%	*P* value
Hours of TV												
≤2	—	—	—	—	—	—	—	—	—	—	—	—
>2	0.21	−0.09–0.52	0.17	0.09	−0.01–0.19	0.07	0.09	−0.06–0.25	0.23	0.03	−0.00–0.05	0.05
Physical activity time (hours)^1^												
>1	—	—	—	—	—	—	—	—	—	—	—	—
≤1	0.23	−0.08–0.55	0.15	0.16	0.05–0.26	**<0.001**	0.08	−0.08–0.24	0.32	0.03	−0.00–0.06	**0.04**
Light physical activity time (hours)^2^												
≤1	—	—	—	—	—	—	—	—	—	—	—	—
>1	−0.04	−0.36–0.28	0.81	0.12	0.01–0.22	**0.02**	0.08	−0.07–0.24	0.29	−0.00	−0.03–0.02	0.69
Sports activity												
Yes	—	—	—	—	—	—	—	—	—	—	—	—
No	−0.43	−1.00–0.13	0.13	−0.10	−0.29–0.08	0.29	−0.04	−0.33–0.23	0.74	−0.04	−0.09–0.01	0.12
Energy balance												
Not positive	—	—	—	—	—	—	—	—	—	—	—	—
Positive	−0.12	−0.48–0.24	0.50	−0.06	−0.18–0.06	0.31	−0.04	−0.22–0.14	0.65	−0.01	−0.04–0.02	0.44

BMI = Body Mass Index; *β* = linear regression coefficient, IC = confidence interval; cm = centimeter; g = gram; TV = television; ^1^play ball, ride bikes, run, and so forth; ^2^play dolls, cars, house, homework, and so forth; ^*^in logarithm.

**Table 7:** Final model of multiple linear regression analysis for the Body Mass Index by age, body fat percentage, percentage of fat in the android region, and waist circumference between 4 and 7 years of age, according to weight gain in the first six months of life, Viçosa, MG, 2011-2012.

Variables	Speed of weight gain (g/day)
BMI/I (escore-*z*)	
*β*	0.10
IC 95%	0.07–0.13
*P* value^1^	**<0.001**
Body fat (%)^*^	
*β*	0.03
IC 95%	0.02–0.04
*P* value^2^	**<0.001**
Fat in the android region (% )^*^	
*β*	0.03
IC 95%	0.02–0.05
*P* value^3^	**<0.001**
Waist circumference^*^ (cm)	
*β*	0.01
IC 95%	0.00-0.01
*P* value^4^	**<0.001**

*β*  = linear regression coefficient; IC = confidence interval; cm = centimeter;  g = gram; Log = logarithm; BMI = Body Mass Index; ^*^in logarithm.

^1^Adjusted for pregestational BMI, gestational weight gain, birth weight, birth length, gender, actual age, place of residence, daily time watching TV, daily time being physically active, sports, weekly frequency for consumption of candies and gums, and weekly consumption of cookies.

^2^Adjusted for pregestational BMI, gestational weight gain, gestational smoking, gender, actual age, daily time watching TV, daily time for physical activity, daily time for light activity, and weekly consumption of cookies.

^3^Adjusted for number of prenatal visits, pregestational BMI, gestational weight gain, gender, actual age, weekly consumption of fried foods, and weekly consumption of cookies.

^4^Adjusted for number of prenatal visits, pregestational BMI, gestational weight gain, birth weight, birth length, gender, actual age, parental education, daily time watching TV, daily time for physical activity, sports, and weekly consumption of cookies.
